# Seeking a New Paradigm for Alzheimer's Disease: Considering the Roles of Inflammation, Blood-Brain Barrier Dysfunction, and Prion Disease

**DOI:** 10.1155/2017/2438901

**Published:** 2017-12-05

**Authors:** Mark E. McCaulley, Kira A. Grush

**Affiliations:** ^1^Yampa Valley Medical Associates, Steamboat Springs, CO, USA; ^2^University of Colorado School of Medicine, Aurora, CO, USA

## Abstract

There is no effective etiologic treatment for Alzheimer's disease, nor is there a prophylactic medication which delays or prevents its onset. The lack of an accurate paradigm is undoubtedly related to the lack of effective means of prophylaxis and treatment. The current paradigm of beta amyloid in Alzheimer's brains causing cognitive dysfunction must be modified. Despite failed clinical trials, research continues into amyloid-oriented treatments. The persistence of the amyloid hypothesis/paradigm is an example of anchoring and representativeness heuristics described by Kahneman and Tversky in their classic 1974* Science* paper. Economic factors also contribute to the persistence of this paradigm. Paradigms impact the scientific process by the following: (1) what is studied; (2) the types of questions that are asked; (3) the structure and nature of the questions; (4) the interpretations of research findings. We review the contribution of inflammation, malfunction of the neurovascular unit, and prion disease to Alzheimer's disease manifestations. Any or all of these are candidates for inclusion into a more accurate, inclusive, and useful new paradigm. By incorporating emerging facts and understanding into a new paradigm, we will enhance our ability to move toward effective prophylaxis and therapy for this tragic disease.

## 1. Introduction

Solanezumab, another promising drug, shows no significant benefit in an Alzheimer's disease clinical trial, announced November 23, 2016.

Just over 110 years ago, Aloysius Alzheimer publicized clinical and pathological findings of the dementing disease which bears his name [1]. From that time through the present, extensive research has yet to determine the underlying cause of the Alzheimer's disease. We can describe the abnormalities, the amyloid plaques and tau tangles, and the intense inflammation. We can list risk factors. However, the underlying cause of the dementia and associated findings remains unknown and unarticulated [2]. Despite intense efforts and economic motivation, a pharmaceutical agent active in preventing the onset or progression of the disease has not been found. Perhaps it stands to reason that, in a disease of unknown etiology, an agent that has an impact on that unknown etiology does not exist.

However, ongoing investigations and promising new ways of understanding the puzzle pieces of Alzheimer's disease have emerged [3]. We propose seeking a paradigm that is consistent with what is known about Alzheimer's disease and offer etiologic probabilities that may inform understanding and suggest research directions in Alzheimer's disease. The purpose of this paper is to explain these developments, providing evidence supporting this progression toward a new paradigm.

## 2. The Amyloid Hypothesis

Beta amyloid accumulates in the brains of Alzheimer's disease patients, forming “senile plaques.” Amyloid plaques are found in most Alzheimer's disease patients, but also in a considerable number of normal individuals [4]. There is substantial evidence supporting the neurotoxicity of beta amyloid, yet therapeutic means to limit the production of amyloid or facilitate its removal have failed to produce clinically meaningful improvement [5]. Removal of beta amyloid from the brain via monoclonal antibody treatment has been associated with brain edema in randomized clinical trials [6]. A 2016 paper reports evidence of beta amyloid providing protection from infection in mouse and worm models of Alzheimer's disease [7].

It seemed a reasonable guess that beta amyloid is involved in causation of most Alzheimer's disease manifestations. Yet, despite the development and clinical trialing of multiple agents that remove beta amyloid from the brain or prevent its production, no US FDA approvals have been granted for such agents. Consistent with the known neurotoxicity of beta amyloid, the clinical trials of the antiamyloid agents have shown some limited neurocognitive benefit, but also neurocognitive worsening. These results suggest that the presence of beta amyloid in the brains of Alzheimer's disease patients is not the primary cause of the disease, but a downstream response to injury, with both beneficial and injurious properties.

## 3. The Inflammation Hypothesis

Alzheimer's disease brains are inflamed brains [8, 9]. Aloysius Alzheimer himself, in his now famous 1907 paper, describes inflammatory cells (microglia) surrounding amyloid plaques in the Alzheimer's disease brain [10].

Initially, the hypothesis of inflammation as a cause of Alzheimer's disease was discarded due to the “immunologic privilege” resulting from the blood-brain barrier. The concept of immune isolation has morphed to an understanding that the brain has unique immunologic properties but is by no means isolated immunologically. There is evidence that brain inflammation may begin in the preclinical stages of Alzheimer's disease [11–17]. Several chemokines and chemokine receptors have been identified in association with Alzheimer's pathologic changes [18]. Complement activation and associated inflammation are characteristic of Alzheimer's disease [19].

Understanding that Alzheimer's disease is consistently associated with inflammation, perhaps early in its course, has led to interest in evaluating methods of controlling or reducing inflammation as a means to prevent or treat Alzheimer's disease, such as nonsteroidal anti-inflammatory drugs (NSAIDs). A considerable number of epidemiologic investigations have provided intriguing insights into the possibility that NSAIDS and other agents may prevent or even treat Alzheimer's disease. A number of these investigations suggested some benefit to their use [20–22]. Yet, randomized controlled trials have shown limited or no benefit [23–27]. We do not accept that lack of benefit of NSAIDs in current randomized controlled trials proves that inflammation is not central to Alzheimer's disease etiology. We have, in a previous paper, discussed problems with the methods utilized in NSAID RCTs [3]. We note the heterogeneity in clinical findings, risk factors, and pathologic characteristics in Alzheimer's disease. However one constant remains:

*Alzheimer's disease brains are inflamed brains*.

## 4. The Neurovascular Unit and Blood-Brain Barrier Breakdown in Alzheimer's Disease

The neurovascular unit is a term inclusive of the blood-brain barrier which modulates the entry of various substances into the brain, thereby regulating the delivery of energy metabolites and preventing the entry of neurotoxic substances into the central nervous system. The neurovascular unit is also comprised of a variety of closely related vascular cells, glial cells, and neurons responsible for regulating the interface between the central nervous system and the peripheral circulation, with both secretory and transport functions. These functions provide a route of egress of toxic substances such as beta amyloid and others from the brain. An intact neurovascular unit, therefore, is essential for appropriate function of the central nervous system.

In the normal aging brain, blood-brain barrier breakdown occurs first in the hippocampus [28]. Significant hippocampal volume loss with associated dysfunction is a fundamental characteristic in early Alzheimer's disease brains and is more pronounced in individuals carrying the APOE4 allele [29]. In a 2015 paper, Montagne et al. presented data suggesting that hippocampal blood-brain barrier dysfunction is an early event contributing to cognitive impairment [28]. In 2016, van de Haar et al. compared 16 early Alzheimer's disease patients with 17 normal controls, showing statistically significant increases in blood-brain barrier leakage in the Alzheimer's disease group [30].

Blood-brain barrier damage may be disruptive or nondisruptive. The former indicates loss of barrier function, and the latter includes damaged transport or secretory mechanisms, which may allow buildup of toxic substances such as beta amyloid, as well as inadequate nutrition.

Systemic inflammation, such as that caused by infections or systemic inflammatory states, damages the blood-brain barrier. TNF alpha and interleukin-1beta are produced in the periphery in sepsis. TNF alpha is transported into and creates damaging inflammation in the brain and blood-brain barrier via upregulation of blood to brain transport mechanisms in the blood-brain barrier. Evidence supporting such damage includes elevated CSF protein levels in sepsis. A damaged blood-brain barrier is more vulnerable to subsequent insults [31].

In a 2016 paper, McArthur et al. review the blood-brain barrier in Alzheimer's disease noting that Annexin A1 seems to promote integrity of the blood-brain barrier [32]. Annexin A1 is elevated in Alzheimer's disease, perhaps as an attempt to repair the faulty blood-brain barrier. Obesity, diabetes, and multiple sclerosis are all characterized by a disrupted blood-brain barrier with evidence for lower levels of Annexin A1 expression.

Pericytes are a type of mural cell found in the microvessels in the brain. Within the neurovascular unit, pericytes are located between endothelial cells, astrocytes, and neurons, making them key players in coordination within the neurovascular unit. Among their chief responsibilities, pericytes are responsible for regulating blood-brain barrier permeability. Pericytes malfunction with aging, resulting in ischemia and increased blood-brain barrier permeability, allowing for the accumulation of toxic substances over time.

In order to examine the role pericytes play in maintaining blood-brain barrier integrity over time, Bell et al. (2010) used adult viable pericyte-deficient mice to demonstrate that pericyte damage or loss does indeed result in a progressive age-dependent vascular-mediated neurodegeneration, and it accomplishes this via two simultaneous pathways: (a) absence of pericytes in adult mice reduced brain microcirculation, diminishing cerebral blood flow that would otherwise mediate chronic perfusion stress and hypoxia, and (b) absence of pericytes in adult mice caused blood-brain barrier breakdown leading to the accumulation of serum proteins and vasculotoxic and neurotoxic macromolecules within the brain.

The same study also examined the role of neuroinflammation as it relates to pericyte damage or loss and found that 16-month-old pericyte-deficient mice showed a significant 2.5-fold increase in the number of activated microglia in the brain and a significant increase in the expression of several inflammatory cytokines—including TNF alpha, interleukin 1-beta and 6, monocyte chemoattractant protein-1, and intercellular adhesion molecule 1—which have been extensively studied and linked to degeneration of Alzheimer's disease brain [33]. In a 2017 paper, Kisler et al. present evidence that pericyte degeneration leads to blood flow disruption, uncoupling of neurovascular responses, brain hypoxia, metabolic stress, and neurodegeneration [34]. In a 2016 paper, Halliday et al. present evidence of blood-brain barrier damage in Alzheimer's disease, with increased fibrin and IgG deposition in Alzheimer's disease brains as a result. The presence of APOE4 allele(s) “leads to accelerated pericyte loss and enhanced activation of LRP1-dependent CypA-MMP-9 blood-brain barrier-degrading pathway in pericytes and endothelial cells, which can mediate greater blood-brain barrier damage” [35].

Of the cells comprising the neurovascular unit, pericytes are the most sensitive to TNF alpha, which causes release from the pericytes of inflammatory mediators to high levels [36]. By controlling blood-brain barrier integrity, pericytes control the movement of peripheral immune cells into the CNS. They have also been shown to engage in phagocytic activity associated with the clearance of unwanted toxic proteins in the CNS. Immunomodulators may elicit the release of inflammatory mediators directly from pericytes while other modulators, such as interferon gamma, impair pericyte function leading to further increase the dysfunction of the blood-brain barrier [37].

A damaged neurovascular unit with abnormal permeability and impaired transport and secretory functioning has the potential to emerge as one of the abnormalities or perhaps* the central* abnormality leading to chronic neurodegenerative disease, particularly Alzheimer's disease. Ongoing research may clarify the role of the neurovascular unit in these diseases.

## 5. Prion Disease

Prions are misfolded proteins that are able to transmit diseases and other characteristics attendant to those proteins, similar to infectious agents. Although viewed chiefly as a cause of disease and disability, prions may play a more fundamental role in physiology or even evolutionary biology as agents that participate in not only pathophysiology, but also normal and desirable physiologic processes [38]. Evidence has been accumulating that prions are involved in neurodegenerative disease in general and Alzheimer's disease in particular. In 2016, Woerman et al. presented evidence of tau based prions propagating in cell culture from Alzheimer's disease and chronic traumatic encephalopathy patients [39]. In 2017, Pandya et al. showed prion-like misfolded proteins spreading via the brain's fiber connectivity network in Alzheimer's disease [40].

Walker, in 2016, presents the case for misfolded proteins playing a role in neurodegenerative diseases with phenotypic variation as a consequence of a variety of factors, such as genetic, epigenetic, and local factors [41]. Misfolded proteins (proteopathic strains) are a part of neurodegenerative diseases but have phenotypic variation induced by a variety of factors.

In a 2015 paper, Jaunmuktane et al. reported apparent human transmission of Alzheimer's disease type amyloid beta pathology with cerebral amyloid angiopathy [42]. Alzheimer's disease appears to be transmissible, suggesting prion-like properties.

Transmission of tau protein prion-like abnormalities has been described in genetically modified laboratory animals [43]. In a 2016 paper, Holmes and Diamond described the prion-like properties of amyloid beta, tau, and alpha-synuclein demonstrating that amyloid, tau, and alpha-synuclein have essential properties of prions [44]. The prion-like properties of alpha-synuclein contribute to interneuronal spread of misfolded proteins [45].

In a 2016 paper, Khanam et al. noted that a variety of neurodegenerative diseases are associated with “massive deposition of misfolded proteins” [46]. A 2017 paper echoes that observation with the statement, “The most common neurodegenerative diseases, such as Alzheimer's, Parkinson's, and amyotrophic lateral sclerosis, are all protein-misfolding diseases” [47].

Hyperphosphorylation of tau, a well-known pathologic mechanism, is shown to impart prion-like characteristics to tau [48]. In 2017, Kriegel et al. presented a paper noting evidence of chronic traumatic encephalopathy being a degenerative tauopathy of hyperphosphorylated tau, behaving as a prion [49].

It is safe to conclude that a large volume of current research indicates that misfolded proteins, more commonly known as prions, are commonly, if not ubiquitously, associated with Alzheimer's disease and other degenerative neurologic diseases.

## 6. A New Paradigm?

“Almost always the men who achieve these fundamental inventions of a new paradigm have been either very young or very new to the field whose paradigm they change. Each paradigm will be shown to establish more or less the criteria that it dictates for itself and to fall short of a few of those dictated by its opponent.”

Thomas S. Kuhn,* The Structure of Scientific Revolutions (1962)*

Of what use is a paradigm? Is it time to shift paradigms in our view of Alzheimer's disease? Philosopher Thomas Kuhn first used the term “paradigm” for science. He stipulated that a paradigm impacts the scientific process by four means:

1. What is studied.
2. The types of questions that are asked.
3. The structure and nature of the questions.
4. The interpretations of research findings.

Kuhn felt that a paradigm shift may incrementally build on a prior paradigm but often represents a fundamental change in how a scientific endeavor is viewed [50].

Our current paradigm in understanding Alzheimer's disease is largely that of the “amyloid hypothesis.” That paradigm has been tested through methods of limiting the production of beta amyloid (and the related tau protein) in or facilitating its removal from the brain. In both cases, the clinical benefits have been nonexistent or small and of no clinical use. The persisting acceptance of the amyloid hypothesis appears to be an example of the anchoring heuristic described by Noble award winning economists/psychologists Tversky and Kahneman in a 1974 Science paper [51]. The persisting acceptance of the amyloid hypothesis may also be an example of the representativeness heuristic described by Kahneman in 2011 [52].

Another rationale for the persistence of the amyloid hypothesis is an economic one. In our current era of market oriented pharmaceutical development, the costs of bringing to clinical trials and eventual approval of a new drug exceed one billion US dollars. The level of investment lost is so great that a considerable reluctance exists to abandon a drug or a paradigm [53, 54]. Investors in the failing drug may be reluctant to abandon a billion dollars' worth of research in an agent that has failed to demonstrate clinical utility.

The lack of a cohesive and accurate paradigm in Alzheimer's disease leads to the lack of etiologically effective medications and treatments. All stakeholders continue waiting for an effective approach to its treatment.

## 7. Which Paradigm?

In seeking to explain an unexplained disease, there are characteristics we should require of our explanation.

1. Persons with the condition being studied should all or nearly all have the proposed explanatory factor, as noted by Kuhn in his seminal 1962 book. The higher the percentage of the afflicted persons having that factor, the higher the probability that the proposed explanatory factor is central to the cause of the condition.
2. The proposed explanatory factor requires plausibility. This necessarily subjective factor is likely to be a source of controversy.
3. The explanatory factor(s) may be plural.

A thorough look at the epidemiologic and randomized controlled trial data of inflammation combatting methods and agents indicates that inflammation as a, or* the*, cause of Alzheimer's disease has not been disproven. On the contrary, the evidence supports the plausibility that inflammation is the key, and methods to limit it may finally slow down or stop the onset and progression of Alzheimer's disease.

Blood-brain barrier abnormalities are very common in Alzheimer's disease brains. Despite the burgeoning data developing on the neurovascular unit, we do not have enough information to know the prevalence of neurovascular unit abnormalities in Alzheimer's disease brains. If an abnormal neurovascular unit is an, or* the*, explanatory factor, it should be present in nearly all Alzheimer's disease brains. Additionally, neurovascular unit abnormalities may cause or be caused by inflammation. Research already done suggests a neurovascular unit-amyloid interaction. TNF alpha may lead to neurovascular unit abnormalities. Subsequent research may illuminate the relative importance of the neurovascular unit and its interactions with other candidate explanatory factors.

Prion characteristics of brain proteins are being studied intensively. Protein folding abnormalities are being found in many Alzheimer's disease brains. As with neurovascular unit abnormalities, the prevalence of prion or other protein folding abnormalities in Alzheimer's disease brains remains to be reported. Similar to our speculation of neurovascular unit dysfunction, inflammation, and amyloid-tau interactions, prions may plausibly impact each of these other potential explanatory factors.

We have limited the above discussion to just a few abnormalities in Alzheimer's disease brains, focusing on characteristic Alzheimer's disease associated abnormalities that hold what we consider the most promise in moving the process forward of finding a useful etiologic explanation. We recognize a number of additional factors impacting the risk of Alzheimer's disease, including genetic, immunologic, environmental, behavioral, toxicological, and psychologic factors that may inform this discussion of new paradigms. We anticipate the possibility that these factors could impact Alzheimer's disease etiology through causing abnormalities in other potential explanatory factors (e.g., inadequate biome diversity increasing propensity for inflammation) [55]. They may also stand on their own despite our subjugating them to an apparent secondary role. There are multiple other factors, little considered by us or others, that could prove pivotal. Examples of such factors could include magnesium intake in Alzheimer's disease-susceptible versus non-Alzheimer's disease-susceptible populations or sleep disorders [56–59].

We believe that a paradigm shift in Alzheimer's disease is warranted. The fact that 100% of Alzheimer's disease brains are inflamed suggests a probability that inflammation is fundamental in the pathophysiology of Alzheimer's disease. One may reasonably anticipate that we will find that inflammation, neurovascular unit dysfunction, beta-amyloid-tau factors, and prion disease interact with one another. Each may amplify the others.

We may find other potential etiologic mechanisms that will need to be incorporated into our view of Alzheimer's disease and other neurodegenerative diseases.

We must, however, recognize the inadequacies in the previous paradigm and move beyond the very real intellectual heuristics and economic factors that stand in the way of prompt and efficient progress toward the truth in understanding the personally, societally, and economically devastating neurodegenerative condition we call Alzheimer's disease.
